# MPASL: multi-perspective learning knowledge graph attention network for synthetic lethality prediction in human cancer

**DOI:** 10.3389/fphar.2024.1398231

**Published:** 2024-05-21

**Authors:** Ge Zhang, Yitong Chen, Chaokun Yan, Jianlin Wang, Wenjuan Liang, Junwei Luo, Huimin Luo

**Affiliations:** ^1^ School of Computer and Information Engineering, Henan University, Kaifeng, Henan, China; ^2^ Henan Key Laboratory of Big Data Analysis and Processing, Henan University, Kaifeng, Henan, China; ^3^ College of Computer Science and Technology, Henan Polytechnic University, Jiaozuo, Henan, China

**Keywords:** synthetic lethality prediction, knowledge graph, multi-perspective learning, deep learning, attention mechanism

## Abstract

Synthetic lethality (SL) is widely used to discover the anti-cancer drug targets. However, the identification of SL interactions through wet experiments is costly and inefficient. Hence, the development of efficient and high-accuracy computational methods for SL interactions prediction is of great significance. In this study, we propose MPASL, a multi-perspective learning knowledge graph attention network to enhance synthetic lethality prediction. MPASL utilizes knowledge graph hierarchy propagation to explore multi-source neighbor nodes related to genes. The knowledge graph ripple propagation expands gene representations through existing gene SL preference sets. MPASL can learn the gene representations from both gene-entity perspective and entity-entity perspective. Specifically, based on the aggregation method, we learn to obtain gene-oriented entity embeddings. Then, the gene representations are refined by comparing the various layer-wise neighborhood features of entities using the discrepancy contrastive technique. Finally, the learned gene representation is applied in SL prediction. Experimental results demonstrated that MPASL outperforms several state-of-the-art methods. Additionally, case studies have validated the effectiveness of MPASL in identifying SL interactions between genes.

## 1 Introduction

Cancer is a genetic disease caused by the accumulation of multiple mutations resulting from the interaction of internal and external factors (Barabási et al., 2011). Traditional cancer treatments such as chemotherapy often have serious side effects and harm healthy cells (Hanahan and Weinberg, 2011). Synthetic lethality (SL) is a genetic interaction that kills cancer cells selectively without damaging healthy cells (Boone et al., 2007; Hartwell et al., 1997; Iglehart and Silver, 2009). SL offers a tremendous depth of research opportunities for anti-cancer drug development and targeted cancer therapy, with researchers making great efforts to identify SL pairs. Discovering SL gene pairs relies heavily on high-throughput wet-lab screening techniques including RNAi screening (Bartz et al., 2006; Luo et al., 2009; Gregory et al., 2010; Blank et al., 2013; Chang et al., 2016) and CRISPR screening (Han et al., 2017; Shen et al., 2017). However, lab experiment-based screening methods are time-consuming and expensive and increase the risk of off-target effects (Liu et al., 2019). Thus, there is an urgent need for efficient and economical methods to overcome the deficiencies of high-throughput screening techniques (Huang et al., 2019).

To overcome these limitations, a several computational methods have been developed for SL prediction. These methods fall into two categories: (i) knowledge-based methods and (ii) supervised machine-learning methods (Zhu et al., 2023). Knowledge-based methods rely on prior knowledge or assumptions (i.e., gene mutations (Lu et al., 2020) or CNVs (Lu et al., 2018)) to detect SL pairs. For example, Zhang et al. (Zhang et al., 2015) proposed a combination of data-driven models with signaling pathway knowledge to discover SL interaction pairs by simulating the effects of gene knockout on cell death. Srihari et al. (Srihari et al., 2015) used copy-number and gene expression data to identify SL interactions. However, knowledge-based methods do not comprehensively utilize underlying patterns of known SL interactions. Machine learning methods such as decision trees (Wong et al., 2004), support vector machines (Paladugu et al., 2008; Qi et al., 2008), random forests (Das et al., 2019), and ensemble classifiers (Pandey et al., 2010; Wu et al., 2014) expedite the identification of SL pairs are challenging to apply to large-scale data due to the complex matrix operations.

Tremendous developments in deep learning-based methods have shown them to be effective in many biomedical tasks, including drug-target prediction (Mohamed et al., 2020), drug-disease prediction (Yu et al., 2021) and drug synergy prediction (Zhang et al., 2023) along with successful applications in SL prediction (Huang et al., 2019; Liu et al., 2019; Cai et al., 2020; Liany et al., 2020; Hao et al., 2021; Long et al., 2021). For example, Long et al. (Long et al., 2021) proposed a graph contextualized attention network to predict SL interactions. This model deploys a dual-attention mechanism to capture the importance of neighbors and feature graphs for node representation learning. Cai et al. (Cai et al., 2020) modeled SL interactions as a graph and adopted a dual-drop GNN to address the sparsity of SL networks. However, most of these methods are limited in the expressive capacity of homogeneous graphs.

Knowledge graphs (KGs) are multi-relational heterogeneous graphs where the nodes and edges correspond to different types of entities and relations, respectively (Wang et al., 2017; 2019b). They overcome the limitations of homogeneous graphs by using rich semantic information between graph entities to discover potential relations. These have begun to equip bioinformaticians with powerful weapons for combining heterogeneous data plainly for SL pairs prediction. Wang et al. (Wang et al., 2021) presented a KGNN-based model, KG4SL, to predict SL interactions. It uses independent knowledge embeddings to capture the underlying biological mechanisms of interconnected SL pairs. Zhu et al. (Zhu et al., 2023) utilized relations in knowledge graphs to represent SL-related factors and learned latent representations of genes through message aggregation. It is evident that employing KG entities such as gene, pathway and their neighbors yields a more accurate embedding representation, but previously KG-based methods ignore the preferences of existing SL interactions and layer-wise differences of entities.

To solve these problems, we develop a novel end-to-end SL prediction model, MPASL, based on multi-perspective learning knowledge graph attention network. Our model consists of four main modules. First, we find gene neighbors via KG hierarchy propagation. Second, KG ripple propagation exploits existing SL interactions preferences to obtain gene representations with finer granularity. Third, MPASL enhances gene representations through a mixed perspective of gene-entity and entity-entity interactions. Specifically, in gene-entity interaction, the knowledge graph relation attention mechanism is designed to score and aggregate gene-oriented entity embeddings to characterize the importance of relationships and informativeness for each entity. Then, the entity enhancement layer obtains the gene-oriented entity embeddings by aggregating the embedding representations of entities and genes. Subsequently, in entity-entity interaction, the discrepancy contrastive layer refine entity embeddings by comparing the various layer-wise neighborhood features of entities, and the attention aggregator obtains the final gene embedding representations by assigning different weight coefficients to the entities. Finally, the objective function using the embedded representation of genes is defined to obtain the predictive scores for unobserved SL pairs.

The contributions of this work are described as follows.

• We propose a novel end-to-end KG-based framework named MPASL, which synthetically and effectively uses ripple propagation and a mixed perspective of gene-entity and entity-entity interactions to learn gene embeddings in the KG.
• To capture the preferences of existing SL interactions and discover potential hierarchical interests of genes, we introduce ripple propagation, which helps to rationally extend the potential interactions of genes and enrich the representation of genes.
• Considering the layer-wise differences between entities, a mixed perspective module obtains a more informative representation of genes from entity-entity perspective by comparing the layer-wise entity embeddings gained from gene-entity perspective learning.
• Comprehensive *in silico* experiments on SynLethDB dataset demonstrate that our MPASL model consistently outperforms other state-of-the-art methods.

The remainder of this paper is organized as follows. The proposed method and the dataset we used are presented in Section 2. Section 3 presented the results and discussion, and Section 4 concluded the paper and discussed the further work.

## 2 Materials and methods

In this section, we introduce the MPASL model. First, we discuss the SL prediction problem. Second, we introduce the dataset used by our model. Then, we provide a detailed explanation of the MPASL model framework and its components. Finally, we discuss the predictions of SL made by the MPASL model.

### 2.1 Problem formulation

We model the SL interactions using an SL graph represented by $G_{\mathrm{SL}}=(V,E)$ , where *V* represents a set of genes, |*V*| is the number of genes involved in SL pairs, and *E* denotes a set of interactions between SL pairs. We use a matrix *A* ∈ {0,1}^|*V*|×|*V*|^ to represent the adjacency matrix of the SL graph. In this adjacency matrix, if there is an SL interaction between Gene *m* and Gene *n*, then *A*_*mn*_ = 1 and 0 otherwise.

In addition to the synthetic lethality between a pair of genes, we consider the auxiliary information of the genes and other related entities in the form of a knowledge graph. The knowledge graph, SynLethKG, is modeled as a heterogeneous graph, with nodes representing diverse entities and edges capturing the relationships between these entities. SynLethKG is represented by $G_{\mathrm{KG}}=(N,E)$ , where N corresponds to a set of nodes of an entity, E∈N\times R\times N represents the set of interactions from the set of relations *R* in the KG between two entities in *N*. Each edge is modeled as a triple T = {(h,r,t)|h,t∈N,r∈E} of entities and relations. For an entity-relation-entity triplet, *h*, *r*, and *t* denote the head entity, relationship, and tail entity of the triple, respectively, with head entities h∈N , tail entities t∈N , and relation entities r∈E . For example, (Lung adenocarcinoma, Associates, *SMAD7*) indicates that *SMAD7* is associated with lung adenocarcinoma (Yeung et al., 2016; Dai et al., 2020). In the graph, nodes represent entities and edges represent relationships from the head entity node to the tail entity node.

Given the SL graph $G_{\mathrm{SL}}$ and the KG of synthetic lethality $G_{\mathrm{KG}}$ , the task is to predict whether there exists a synthetic lethal relationship between genes *m* and *n*. This is done by learning a mapping function ${\hat{y}}_{\mathrm{mn}}=F(m,n;\Theta ;G)$ , which automatically generates gene embeddings from SynLethKG $G_{\mathrm{KG}}$ and estimates the probability of SL interaction between gene *m* and *n* in the SL graph $G_{\mathrm{SL}}$ to identify potential SL pairs, where Θ represents the weight parameter of the model function F .

### 2.2 Dataset description

SynLethDB (Wang et al., 2022) is a comprehensive and up-to-date database that containing information on SL interactions. It collects SL gene pairs from various sources, including biochemical analysis, public databases (Schmidt et al., 2013; Oughtred et al., 2019),computational predictions (Ryan et al., 2014), and text mining. It covers SL gene pairs in humans and four model organisms (mice, fruit flies, worms and yeast) and a gene-related knowledge graph called SynLethKG, which comprises 11 types of entities and 24 types of relationships. SynLethKG collects a variety of relationships for genes involved in synthetic lethal gene pairs, including gene-compound associations, gene-cancer associations, and other features about genes, drugs, and cancers, such as (Anatomy, expresses, gene), (Disease, presents, symptom) and (Gene, regulates, gene). In addition, 7 out of the 11 types of entities are directly related to genes, namely, anatomy, biological process, cellular component, compound, disease, molecular function, and pathway. According to (Wang et al., 2021), we used the same synthetic lethality data and knowledge graph as it. The SL gene pairs in SynLethDB have been widely used for training and testing machine learning models for SL prediction. Since the number of negative samples provided by SynLethDB for SL interactions is much less than the number of positive samples, we generated negative samples using the method used in KG4SL (Wang et al., 2021), where an equal number of unknown gene pairs were randomly selected as non-SL gene pairs to balance out the difference in distribution between positive and negative samples. In our study, we specifically focused on human SL interactions. The final SL dataset we used included 72,804 gene pairs involving 10,004 genes. Additionally, the KG used in our study had 54,012 nodes and 2,231,921 edges. Tables 1 and 2 summarize the statistics for SL and SynLethKG. Tables 3 and 4 show detailed information about the entities and relationships of SynLethKG.

**TABLE 1:** Statistical information on SL datasets.

	No.of genes	No.of interactions	Positive pairs	Negative pairs
SL data	10,004	72,804	36,402	36,402

**TABLE 2:** SynlethKG’s statistics.

Datasets	Entity types	Relationship types	No.of nodes	No.of edges
SynlethKG	11	24	54,012	2,231,921

**TABLE 3:** Details of entities in SynLethKG.

Type	No.of entities
Anatomy	400
Biological process	12,703
Cellular component	1,670
Compound	2,065
Disease	136
Gene	25,260
Molecular function	3,203
Pathway	2,069
Pharmacologic class	377
Side effect	5,702
Symptom	427

**TABLE 4:** Details of relationships in SynLethKG.

Type	No.of relationships
(Anatomy, downregulates, gene)	31
(Anatomy, express, gene)	6,17,175
(Anatomy, upregulates, gene)	26
(Compound, binds, gene)	16,323
(Compound, causes, side effect)	1,39,428
(Compound, downregulates, gene)	21,526
(Compound, palliates, disease)	384
(Compound, resembles, compound)	6,266
(Compound, treats, disease)	752
(Compound, upregulates, gene)	19,200
(Disease, associates, gene)	24,328
(Disease, downregulates, gene)	7,616
(Disease, localizes, anatomy)	3,373
(Disease, presents, symptom)	3,401
(Disease, resembles, disease)	404
(Disease, upregulates, gene)	7,730
(Gene, covaries, gene)	62,966
(Gene, interacts, gene)	1,47,638
(Gene, participates, biological process)	6,19,712
(Gene, participates, cellular component)	97,652
(Gene, participates, molecular function)	1,10,042
(Gene, participates, pathway)	57,441
(Gene, regulates, gene)	2,67,302
(Pharmacologic class, includes, compound)	1,205

### 2.3 Framework design

**FIGURE 1:**
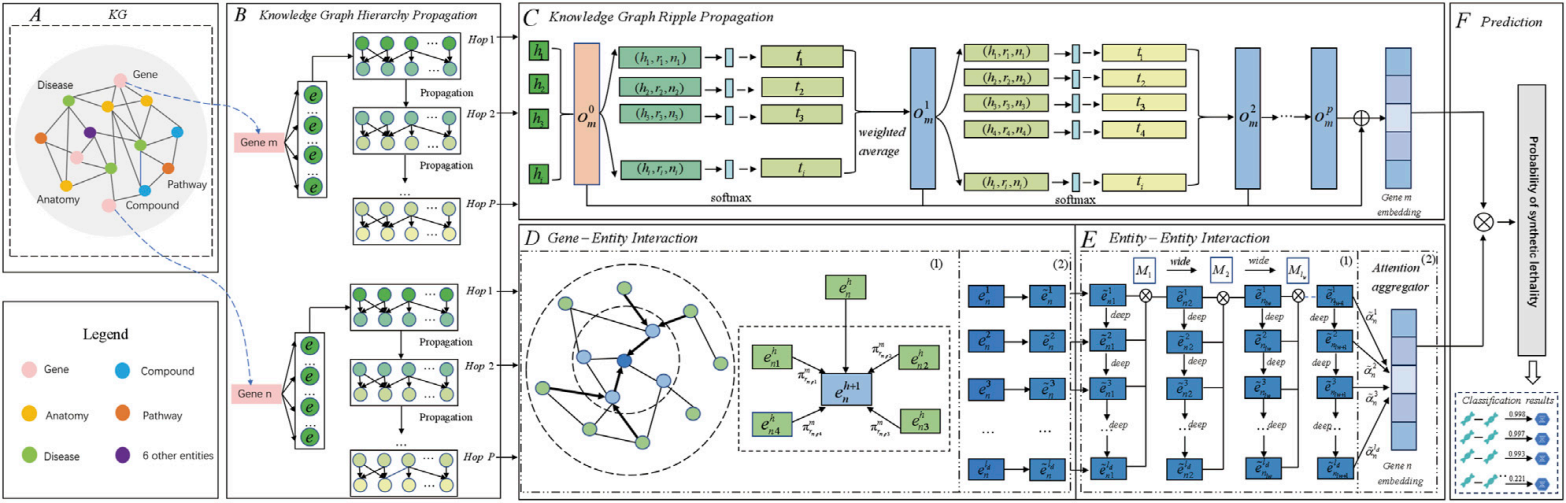
Architecture of MPASL. **(A)** KG. The KG assists SL prediction and consists of 11 kinds of entities and 24 kinds of relationships. **(B)** Knowledge graph hierarchy propagation identifies sets of neighboring nodes for gene entities. Symbol *e* represents the initial entities associated with gene entities, which are sets of tail entities directly related to genes. Multi-hop sets correspond to triples associated with genes. The knowledge-based higher-order interaction information for genes is stored in these multi-hop sets. **(C)** Knowledge graph ripple propagation uses the KG to model gene embeddings at a finer level. **(D)** Gene-entity interaction. D(1) A KG relation attention mechanism applies attention scoring to surrounding relations in a gene-specific manner. D(2) The entity enhancement layer aggregates entity embeddings specific to genes to allocate different amounts of information to refine their embeddings. **(E)** Entity-entity interaction. E(1) Discrepancy contrastive layer integrates different high-order connectivity information for genes in terms of depth and width. E(2) The attention aggregator module employs an attention aggregator to assign different weight coefficients for different genes and generate updated gene embeddings. **(F)** The prediction module outputs the predicted probabilities of synthetic lethality between genes.

The overall pipeline of MPASL is shown in Figure 1. The model consists of four modules including knowledge graph hierarchy propagation, knowledge graph ripple propagation, a mixed perspective of gene-entity and entity-entity interactions module and prediction module. In order to present the article more clearly, a mixed perspective of gene-entity and entity-entity interactions module is divided into two parts: gene-entity interaction and entity-entity interaction.

(1) Knowledge graph hierarchy propagation. This layer maps entities and relationships in the KG to vectors. We then recursively explore the set of multi-source neighbor nodes that are directly or indirectly related to genes in the KG.
(2) Knowledge graph ripple propagation. In this module, we introduce finer-grained entity embedding propagation using the set of existing SL interaction preferences for genes. This recursively extends the representation of genes with supplementary edge information, allowing for the automatic discovery of potential paths from genes with SL interactions to candidate genes. This approach connects the existing SL interaction set of genes with the prediction records, bringing interpretability to SL prediction.
(3) Gene-entity interaction. We split gene-entity interaction into KG relation attention mechanism and an entity enhancement layer. These layers score, aggregate, and update the embeddings of specific genes and entities with their neighborhood information, explicitly capturing the higher-order structural information and similarities in the knowledge graph and contributing to a stable learning process.
(4) Entity-entity interaction. We use a discrepancy contrastive layer to hierarchically compare the connected information of entities across different layers. We also employ an attention aggregator to obtain different weight coefficients for neighborhoods in the mixed perspective of entity. Iteratively propagating and updating entity representations with multiple layers of information increases the diversity of predicted embeddings.
(5) Prediction module. This module illustrates the learning and prediction of SL, using a series of aggregated and updated gene representations to compute prediction scores.

#### 2.3.1 Knowledge graph hierarchy propagation

MPASL’s knowledge graph hierarchy propagation obtains a set of multi-hop neighboring nodes for a set of genes. This layer encodes the crucial hierarchical information into the gene representations, enriching those representations constructed from entities in the KG and including the existing set of SL interactions.

The rich semantic connections between entities in the KG help identify potential complex relationships between entities. These complex relationships provide an additional perspective for exploring SL genes, aiding in the discovery of potential connections between genes and improving the accuracy of SL prediction. Obtaining relevant gene information from the KG requires information of associated entities having highly correlated relationships. Essentially, the entities having SL relationship with a gene provide at least some information about gene attributes. By transforming and comparing genes with entities, turning the related entity set obtained from existing SL interactions into an initial seed set for propagation in the KG, we capture information on gene-gene interactions. With the initial seed set, we can propagate KG associations from near to far along the KG, obtaining an extended entity set and a triple set of *p*-hops, effectively enriching the potential vector representation of genes. In summary, modeling gene representations by using relevant entities in the KG enhances gene information.

To do all of this, we first define the extended entity set of genes. For the input gene *o*, the set of entities with SL interactions with that input gene is treated as seeds in the KG. Then it extends along the KG to form a set of p-hopped extended entity sets $\varepsilon_{o}^{p}$ of the gene *o*, effectively expressing the interaction information of the potential semantics of the entities. The adjacent entity sets of gene *o* can be recursively represented as:

$$\varepsilon_{o}^{p}=\left\{t|\left(h,r,t\right)\in G\;and\;h\in \varepsilon_{o}^{p-1}\right\},\;p=1,2,\ldots ,l_{p}$$ (1)

where *p* represents the distance from the initial set of entities. $\varepsilon_{o}^{0}=\left\{o|y_{mn}=1\right\}$ is the initial set of genes having SL relationships with gene *o* and serves as the seed set of gene *o* in the KG. This design emphasizes the original information of genes and reduces biases caused by multiple propagation layers, making it more effective in expanding potential vector representations of entities.

For a central gene in a KG subgraph, the set of entities $\varepsilon_{o}^{0}=\left\{o|y_{mn}=1\right\}$ that already have synthetic lethality relationships with this gene is regarded as the starting point in the KG. The set of *p*-hopping triplet propagation constructed with this starting point is explored along the KG relationship:

$$S_{o}^{p}=\left\{\left(h,r,t\right)|\left(h,r,t\right)\in G\;and\;h\in \varepsilon_{o}^{p-1}\right\},\;p=1,2,\ldots ,l_{p}$$ (2)

It is meaningful to construct the model using knowledge graphs as edge information, as adjacent entities can be seen as intuitive extensions of gene features. The knowledge graph extends the neighboring nodes in each layer, propagating layer by layer, from near to far, effectively capturing high-order interactive information based on the KG through hierarchy propagation. Symbolically, *ɛ* consists solely of tail entities, *S* is a set of knowledge triplets, *p* represents (one or more) hops, and *l*_*p*_ is the number of hops. To reduce the computational burden of MPASL, we use a fixed-size set of neighbors (Wang et al., 2019b) for each entity instead of the complete neighbor set.

#### 2.3.2 Knowledge graph ripple propagation

We extend gene representations by supplementing auxiliary information with a KG ripple propagation to model interactions between genes in a finer grained manner. This technique relies on traversing all relevant entities and associations along ripple propagation in the KG. This process recursively captures the topological neighborhood structure of the central entity in multi-hop ripple sets. This helps to expand potential preference genes, increase the diversity of predicted embeddings, and discover potential SL relationships. When a given tail entity in the KG has different head entities and relationships, it carries different meanings and potential vector representations. The gene representation of the KG ripple propagation is constructed from the gene SL response *O*_*m*_ generated by the triplet propagation set *S*_*m*_ to explore the potential gene relationships.

To perform this operation, we first define the gene potential SL response $o_{m}^{0}$ for the 0-hop based on entity $h_{i}\in S_{m}^{1}$ , where *h*_*i*_ represents the head entity that has existing SL relationship with gene *m* and $S_{m}^{1}$ is the one-hop triplet propagation set of gene *m* obtained from KG hierarchy propagation. Each gene *n* is assigned a different weight towards the SL preference response of gene *m*:

$$o_{m}^{0}=\underset{h_{i}\in s_{m}^{1}}{\sum }a_{i}h_{i}$$ (3)

$$a_{i}=soft\;max_{i}\left(W_{a}\left[h_{i},n\right]\right)$$ (4)

where *W*_*a*_ is a trainable parameter. The vector $o_{m}^{0}$ represents the 0th-order response of known SL interactions of gene *m* with respect to entity embedding *n*. In part C of Figure 1, we use the orange rectangle to represent the 0th hop SL response, and the p-hop (*p* ≥ 1) SL response is represented by the blue rectangle.

Second, apart from the 0th jump, gene embeddings *m* are achieved by adding SL non-zero hop responses. The ripple set $S_{m}^{p}$ is a set of triples that are *p* hops away from the seed set $\varepsilon_{o}^{0}$ . These ripple sets are used to interact with the SL 0th-order response to obtain the *p* hop response of gene *m* to SL. Given the gene embedding *n* and the one-hop triplet propagation set $S_{m}^{1}$ of gene *m*, for each triplet (*h*_*i*_, *r*_*i*_, *t*_*i*_) in $S_{m}^{1}$ , the associated probability is assigned by comparing gene *n* with the head entity *h*_*i*_ and relation *r*_*i*_ in the triplet propagation set $S_{m}^{1}$ . Finally, after obtaining the correlation probability *k*_*i*_, and the SL response $o_{m}^{p}$ of gene *m* is calculated as a sum of the weighted tails corresponding to the correlation probability *k*_*i*_. Finally, the vector $o_{m}^{p}$ is returned:

$$o_{m}^{p}=\underset{\left(h_{i},r_{i},t_{i}\right)\in S_{m}^{p}}{\sum }k_{i}t_{i},\;p=1,2,\ldots ,l_{p}$$ (5)

$$k_{i}=soft\;max\left(n^{T}r_{i}h_{i}\right)=\frac{\exp\left(n^{T}r_{i}h_{i}\right)}{\underset{\left(h,r,t\right)\in S_{m}^{1}}{\sum }\exp\left(n^{T}r_{i}h_{i}\right)}$$ (6)

where *p* > 0, $h_{i}\in R^{S},r_{i}\in R^{S\times S}$ are the head entity *h*_*i*_ and relation *r*_*i*_, $t_{i}\in R^{S}$ is the tail entity, and $n\in R^{S}$ is the embedding of gene *n*. In the embedding relationship *r*_*i*_ space, genes and entities may have different similarities under different relationships, and the associated probability *k*_*i*_ can be regarded as measuring the degree of similarity between genes *n* and entities *h*_*i*_ in the space of relation *r*_*i*_.

We repeat the process of KG ripple propagation to obtain the first-order response $o_{m}^{1}$ of genes *m* and the second-order response $o_{m}^{2}$ of genes *m*, and this process can be iteratively performed on the triplet propagation set $S_{m}^{i}$ of gene *m* in *i* = 1, … , *p*. After integrating all gene preference responses $o_{m}^{p}$ , we generate the final embedding of gene *m* by integrating all *p*-order responses:

$$o_{m}=concat\left(\left[o_{m}^{0},o_{m}^{1},\ldots ,o_{m}^{p}\right]\right)$$ (7)

$$m=w_{o}o_{m}+b_{o}$$ (8)

#### 2.3.3 Gene-entity interaction

To capture the high-order similarities between gene-related entities in the KG, we propose a gene-entity interaction module. It consists of two parts: a KG relation attention mechanism and an entity enhancement layer. Each entity in the KG has different neighboring entities and relationships, leading to different meanings and potential vector representations. Furthermore, there exist complex associations among neighboring entities. We construct a weighted subgraph specific to each SL-related gene from the KG, allowing us to focus on the relevant entities. To capture entity embeddings, we apply a KG relation attention mechanism that takes into account the relationships between an entity and its individual neighbors, allowing us to describe the importance of each relationship to a specific entity and provide a more detailed understanding of its context. Additionally, we equip the gene-specific entity embeddings with enhancement operations to stabilize the latent representation of the entity in the embedding space.

##### 2.3.3.1 KG relation attention mechanism

When MPASL collects information from the vicinity of gene *n* in the KG, it scores each relation surrounding gene *n* in a manner specific to gene *m*. Thus, the gene *m*-oriented manner can be viewed as an early layer that increases the interaction of gene *m* with its weighted subgraph center entity *n*, and then the gene-oriented KG relation attention mechanism aggregates neighbor information in a gene *m*-specific manner. For any central entity *n* in the weighted subgraph oriented to gene *m*, different relationships have different indication weights for an entity, and the key step is to identify relevant nodes and determine the weight of edges to avoid assigning the same weight to different neighbors in the process of information aggregation. The weight of each edge is defined by a relation scoring function specific to gene *m*, and the proposed KG relation attention exploiting the information of gene *m*, gene *n*, and the relation to determine which neighboring entity connected to gene *n* is more informative. Therefore, each neighboring entity is weighted by attention *π*, where *m* represents different known genes and *r*_*n*,*e*_ represents the relationship *r* from the entity *n* to the neighboring entity *e*. We aggregate and weight each neighboring node of the entity to generate the final representation n(N(n)) of any central entity in the gene *m*-specific weighted subgraph:

$$n\left(N\left(n\right)\right)=\underset{e\in N\left(n\right)}{\sum }{\tilde{\pi }}_{r_{n,e}}^{m}e$$ (9)

Assuming *n* is the central node, *N*(*n*) is a set of entities directly connected to *n*, and the size of *N*(*n*) can vary greatly among all entities. To maintain efficiency and consistency in each batch calculation mode, we uniformly extract a fixed number of *k* neighbors for each entity to represent its local structure (Wang et al., 2019b), and repeat this process *p* times.

In a subgraph specific to gene *m*, for the SL pair (*m*, *n*), the weight of the edge *r*_*m*,*n*_ is calculated as $\pi_{r_{n,e}}^{m}$ , where *e* is one of the entities specific to the gene *m* subgraph, and *e* ∈ *N*(*n*). In addition, *m* and *r*_*n*,*e*_ are feature embeddings of the gene *m* and relation *r*_*n*,*e*_, and the attention score $\pi_{r_{n,e}}^{m}$ denotes the attention weight of the relation *r*_*n*,*e*_ with respect to the gene *m*. The higher the attention weight, the more important the neighboring entity is, and the more informative the neighboring entity connected to gene *m* becomes. The incorporation of an attention mechanism, enables learning different weights for different neighbors (Veličković et al., 2017). To compute the attention scores of the neighbors in the weighted subgraph *π*, we implement the function $\pi_{r_{n,e}}^{m}$ by means of a neural network similar to the attention mechanism. To generate the final function $\pi_{r_{n,e}}^{m}$ specific to any central entity of the weighted subgraph of the gene *m* we use the following formulas:

$$z_{0}=ReLU\left(W_{1}\left(n\|r_{n,e}\right)+b_{1}\right)$$ (10)

$$\pi_{r_{n,e}}^{m}=\sigma \left(W_{3}ReLU\left(W_{2}z_{0}+b_{2}\right)+b_{3}\right)$$ (11)

where ReLU is the nonlinear activation function, ‖ represents the concatenation operation, and *W* and *b* are the trainable weights and biases. Specifically, *W*_1_ and *b*_1_ in Eq. 10 represent the weight and bias for the first layer of the neural network, while *W*_2_, *b*_2_, *W*_3_ and *b*_3_ denote the weights and biases for the second and output layers in Eq. 11, respectively. The nonlinear activation function *σ* is set as Sigmoid.

To make the attention coefficients among different entities comparable (with the sum of the attention coefficient of all adjacent nodes being 1), we use the softmax function to normalize the coefficients of all entities *e* related to the gene *n* (Veličković et al., 2017). The final attention score highlights the neighboring nodes that should receive more attention to capture the entity embedding. The softmax function can be expressed as:

$${\tilde{\pi }}_{r_{n,e}}^{m}=\pi \left(n,e\right)=softmax\left(\pi \left(n,e\right)\right)=\frac{\exp\left(\pi_{r_{n,e}}^{m}\right)}{\underset{e^{\prime }\in N\left(n\right)}{\sum }\exp\left(\pi_{r_{n,e^{\prime }}}^{m}\right)}$$ (12)

##### 2.3.3.2 Entity enhancement layer

To further enhance the interaction between genes and entities, we propose a gene-specific entity enhancement layer. Previous approaches have neglected the effect of multiple entity embeddings on gene richness and overlooked the comprehensive expression of entities and genes. For different genes, KG entities have different amounts of information to describe their properties. For example, *BNIP3* is a well-known tumor suppressor, while *FTO*, as an N6-methyladenosine RNA demethylase, is upregulated in human breast cancer. It has been observed that *FTO* suppresses cell apoptosis by downregulating *BNIP3* (Niu et al., 2019). Under hypoxic conditions, the mRNA levels of *BNIP3* increase in CHO cell lines, and this effect is mediated by Hif-1*α* (Bruick, 2000). Therefore, the entity enhancement layer aggregates each entity with genes through an aggregation operation to enhance and enrich the entity embeddings. The enhancement function can be either linear or nonlinear:

$$\tilde{e}=W_{e}\left(agg\left(e,m\right)\right)+b_{e}$$ (13)

$$\tilde{e}=\sigma \left(W_{e}\left(agg\left(e,m\right)\right)+b_{e}\right)$$ (14)

where *W*_*e*_ and *b*_*e*_ are the trainable weight matrix and bias, and *agg* is a nonlinear activation function.

In this study, we implemented four types of aggregation methods $agg:R^{S}\times R^{S}\to R^{S}$ are follows:

• Sum Aggregator (Wang et al., 2019b) refers to a process of summing the representation vectors of two entities, and applying a nonlinear transformation to the resulting vector:

$$\tilde{e}=\sigma \left(W\left(e+m\right)+b\right)$$ (15)

• Concat Aggregator (Wang et al., 2019b) combines the representation vectors of two entities before applying a nonlinear transformation:

$$\tilde{e}=\sigma \left(W\cdot concat\left(e,m\right)+b\right)$$ (16)

• Pooling Aggregator (Glorot et al., 2011) calculates the maximum value from multiple vectors within the same dimension and subsequently applies a nonlinear transformation:

$$agg_{\mathrm{pool}}^{\left(e\right)}=\sigma \left(W\cdot pool_{\max}\left(T_{0}\right)+b\right)$$ (17)

• Top-k Aggregator (Kumar et al., 2009) efficiently aggregates information from multiple sorted lists of vectors to compute the top *k* objects:

$$\tilde{e}=Top\text{\_}K\left(\sigma \left(W\left(e,m\right)+b\right),k\right)$$ (18)

The function *Top*_*K* (*data*, *k*) extracts the top *k* data values in order. As shown in Eq. 18, the top *k* values are taken after sorting the vector *σ*(*W* (*e*, *m*) + *b*) in descending order.

#### 2.3.4 Entity-entity interaction

The entity-entity interaction consists of a discrepancy contrastive layer and an attention aggregator. The former focuses on capturing higher-order connectivity between entities, hierarchically comparing layered information to further improve entity embeddings. The latter performs weighted aggregation of embedding to avoid noise caused by excessive node embedding information, which could otherwise affect prediction results.

##### 2.3.4.1 Discrepancy contrastive layer

Our focus is on incorporating the latent information of neighbors at different distances into the information comparison at each layer, capturing higher-order message passing between entities, and enhancing the representation of entity embeddings through the overall differentiation of hierarchical entities. We introduce the hierarchical modeling capabilities of the model in terms of depth and width.

For depth, we integrate the gene ${\tilde{e}}_{n_{w}}^{d}$ and neighborhood information $\tilde{n}{\left(N\left(n\right)\right)}_{w}^{d}$ collected from different depths. By comparing neighbors of different orders in high-order message passing, each node receives potential vector representations from neighboring nodes or further *d*-order neighbors. Then we aggregate them into $agg\left(\cdot \right):R^{S}\times R^{S}\to R^{S}$ to generate the mixed next-order depth embedding ${\tilde{e}}_{n_{w}}^{d+1}$ . Here, we use the Top-*k* Aggregator to aggregate gene representations and their neighborhood information into a single vector.

For width, the feature differences between entities located at different width distances means this wide-layer feature difference plays a critical role. In terms of the training space of the model, entity features at different width levels should be compared (Abu-El-Haija et al., 2019) so that the model can choose potential information by comparing neighbors at various distances. Therefore, we perform a contrastive mixed operation of neighborhood latent features within different width distances. As the relevance of each layer in the network varies, it is possible for entities to be connected to neighboring nodes with different attributes or labels. We use the width-layer matrix *M*_*w*_ to integrate the deep neighborhood information $\left({\tilde{e}}_{n_{w}}^{1},{\tilde{e}}_{n_{w}}^{2},\ldots ,{\tilde{e}}_{n_{w}}^{d}\right)$ with different properties in a layer-wise and progressively deeper manner, updating the high-order embedding representation of wide layer entities ${\tilde{e}}_{n_{w+1}}^{1}$ :

$${\tilde{e}}_{n_{w+1}}^{1}=M_{w}\left(concat\left(\left[{\tilde{e}}_{n_{w}}^{1},{\tilde{e}}_{n_{w}}^{2},\ldots ,{\tilde{e}}_{n_{w}}^{d}\right]\right)\right)$$ (19)

$${\tilde{e}}_{n_{w}}^{d+1}=agg\left({\tilde{e}}_{n_{w}}^{d},\tilde{n}{\left(N\left(n\right)\right)}_{n_{w}}^{d}\right)$$ (20)

##### 2.3.4.2 Attention aggregator module

After *l* rounds of discrepancy contrastive layers, we obtain multiple embedding representations of gene *n*. This module uses the gene *n* representation set $T_{n}=\left\{{\tilde{e}}_{n_{lw}}^{\left(1\right)},{\tilde{e}}_{n_{lw}}^{\left(2\right)},\ldots ,{\tilde{e}}_{n_{lw}}^{\left(i\right)}\right\},\;i=0,1,\ldots ,l$ to update the embedding of gene *n* uniformly. We use an attention aggregator module that assigns different importance levels to each embedding, avoiding giving each embedding the same weight when aggregating information. For the potential features of the gene *n*, the attention aggregator first learns the attention scores for each embedding. Then, the scores are normalized to derive weight coefficients for the embeddings. Finally, the attention aggregator performs a weighted aggregation on all embedding representations to update the embedding of the gene *n*:

$$\alpha_{n}^{\left(i\right)}=w_{6}^{T}\tanh\;\left(W_{6}agg_{\mathrm{pool}}^{\left(e_{n}\right)}\right)$$ (21)

$${\tilde{\alpha }}_{n}^{\left(i\right)}=\frac{\exp\left(\alpha_{n}^{\left(i\right)}\right)}{\overset{T_{0}}{\underset{i^{\prime }}{\sum }}\exp\left(\alpha_{n}^{\left(i^{\prime }\right)}\right)}$$ (22)

$$n=\sigma \left(W_{7}\underset{{\tilde{e}}_{n_{lw}}^{\left(i\right)}\in T_{o}}{\sum }{\tilde{\alpha }}_{n}^{\left(i\right)}{\tilde{e}}_{n_{lw}}+b_{4}\right)$$ (23)

$$agg_{\mathrm{pool}}^{\left(e_{n}\right)}=\sigma \left(W\cdot pool_{\max}\left(T_{n}\right)+b\right)$$ (24)

where tanh is the nonlinear activation function assigned to the prediction model. The parameters $w_{6}\in R^{S}$ and *W*_6_, $W_{7}\in R^{S\times S}$ are weight vector and weight matrices, respectively; $b_{4}\in R^{S}$ is the bias term; and *σ* is the *Sigmoid* activation function.

#### 2.3.5 Synthetic lethality prediction

After obtaining the final potential embeddings of gene *m* and gene *n*, MPASL combines the two latent features through the prediction function *f*_*SL*_ to obtain the final predicted probability that gene *m* and gene *n* are SL relationships, where *f*_*SL*_ is the inner product function. *σ* is the *Sigmoid* function, which compresses the output to the range between 0 and 1, indicating the probability of the SLs:

$${\hat{y}}_{\mathrm{mn}}=\sigma (f_{\mathrm{SL}}(m,n))$$ (25)

### 2.4 Objective function

We now consider the real-valued label function $l_{m}:E\to R$ on the KG, which is constrained to take a specific value *l*_*m*_(*n*) = *y*_*mn*_ at node n∈N⊆E . If gene *m* is found to be relevant to *n*, then *l*_*m*_(*n*) = 1, otherwise *l*_*m*_(*n*) = 0. We use label smoothness to act on the supervised signals of regularized edge weights (Wang et al., 2019a):

$$R\left(A\right)=\underset{m}{\sum }R\left(A_{m}\right)=\underset{m}{\sum }\underset{n}{\sum }J\left(y_{mn},{\hat{l}}_{m}\left(n\right)\right)$$ (26)

where *A*_*m*_ aggregates the representation vectors of neighboring entities. The ideal edge weight matrix *A* should reproduce the true relevance labels of each entity while satisfying the smoothness of relevancy labels. We combine the knowledge-aware graph neural network with least squares regularization and use negative sampling during the training process to optimize MPASL. The complete loss function is obtained as:

$$L=\underset{m\in G}{\sum }\left(\underset{n:y_{mn}=1}{\sum }J\left(y_{mn},{\hat{y}}_{mn}\right)-\overset{N^{m}}{\underset{i=1}{\sum }}E_{N_{i}∼P\left(n_{i}\right)}\;J\left(y_{mn_{i}},{\hat{y}}_{mn_{i}}\right)\right)+\gamma \|F{\|}_{2}^{2}+\lambda R\left(A\right)$$ (27)

where the first term J is the cross-entropy loss, *N* ^*m*^ is the number of negative samples for gene *m*; with *N* ^*m*^ = |{*n*: *y*_*mn*_ = 1}|, and *P* is a negative sampling distribution and follows a uniform distribution. The second term is L2 regularization. The third part *R* (⋅) corresponds to the label smoothness component, which can be viewed as adding the constraint of edge weight *A*. Therefore, *R* (⋅) serves as a regularization on *A* to assist in learning the edge weights. *λ* and *γ* are balance hyperparameters.

## 3 Experiments and results

We compare the performance of the MPASL with several baseline models to comprehensively evaluate its performance. Additionally, we conducted parameter sensitivity analysis and ablation studies to further investigate the model’s performance. The MPASL model was implemented using Python 3.6 and TensorFlow 1.15.0. In the SynLethDB dataset, we split the gene pairs into training, validation, and test sets in a ratio of 7:1:2. We use the area under the ROC curve (AUC) and the area under the precision-recall curve (AUPR) as evaluation metrics to assess the predictive performance. Finally, we present a case study to demonstrate the mechanisms of potential SL interactions between two genes.

### 3.1 Parameter settings

We evaluated our model parameters using 5-fold cross-validation and used a grid search to choose the optimal hyperparameter settings. We tested the following MPASL parameters: batch sizes $\in \left\{32,64,128,256,512,1024,2048\right\}$ , learning rates $\in \left\{6\times 10^{-5},10^{-4},10^{-3},10^{-2}\right\}$ , the entity embedding dimensions $\in \left\{8,16,32,64,128,256,512\right\}$ , the numbers of layers for KG ripple propagation, and the depth and width of discrepancy contrastive layers $\in \left\{1,2,3,4,5\right\}$ , and the ripple preference set sizes $\in \left\{4,8,16,32,64\right\}$ . After these tests, we set the number of KG neighbor samples to 8, initialized the number of embedding dimensions to 128, set the early stopping level to 5 and set the regularization weight 1 × 10^−8^. Table 5 provides hyperparameter settings in detail.

**TABLE 5:** The hyperparameter setting.

Parameter	Setting
Batch size	512
Learning rate	6 × 10^−5^
dim	128
p_hop	2
depth	2
width	3
L2_weight	1 × 10^−8^
LS_weight	1 × 10^−8^
optimizer	Adam
n_samples	8
ripple_set_size	8

### 3.2 Comparison with previous studies

To validate the performance of MPASL, we compared our model with several recently proposed baseline methods for SL prediction. These benchmark methods include SL^2^MF, GRSMF, DDGCN, GCATSL, KG4SL, and SLGNN. It is worth noting that the first four methods do not utilize KGs to generate gene embeddings. We used the default settings specified in their original implementations in our tests. Below are brief descriptions of these comparison methods.

(1) SL^2^MF (Liu et al., 2019) uses logical matrix factorization and further integrates gene similarity based on gene ontology (GO) annotations to predict human SL interactions.
(2) GRSMF (Huang et al., 2019) is a graph regularized self-representation matrix decomposition model predicting SL interactions from regularized graphs of data from different sources.
(3) DDGCN (Cai et al., 2020) predicts sparse SL interactions using dual-dropout graph convolutional networks (GCNs).
(4) GCATSL (Long et al., 2021) performs SL prediction using a graph contextual attention network.
(5) KG4SL (Wang et al., 2021) represents the first novel SL interaction prediction model based on knowledge graphs and graph neural networks, effectively leveraging rich semantic information encoded in KGs.
(6) SLGNN (Zhu et al., 2023) is a factor-aware knowledge graph neural network for learning gene embeddings and predicting SL interactions.

It is also important to address the potential bias that may arise when there are more positive than negative training pairs. In such cases, many prediction algorithms achieve high performance on the test set by simply manipulating the features of each pair. We observed this situation in SL prediction methods as well. Reliable estimation of prediction error is challenging, especially when the model is uncertain and requires independent test subjects. These test subjects must not participate in model construction or model selection. A more effective approach is to utilize stratified nested cross-validation (Preuer et al., 2018), where the test set is selected to exclude synthetic lethality gene pairs, denoted as the “Leave out synthetic lethality” setting. We used a 5-fold nested cross-validation setup in which hyperparameters were selected in the inner loop based on validation error, and then the best performance model for the inner loop was evaluated on the outer test fold to obtain performance estimates that were not affected by hyperparameter selection.

Our model was experimented under two evaluation settings: random cross-validation and stratified cross-validation. The prediction results, denoted as “Random CV” and “Leave out synthetic lethality”, are shown in Table 6. From the AUC and AUPR scores, our MPASL outperformed the other methods. Specifically, on the SynlethDB dataset, the MPASL model achieved an AUC value of 0.9656 and an AUPR value of 0.9798. In the Leave out synthetic lethality setting, the MPASL model achieved an AUC of 0.8766 and an AUPR of 0.8941. These values surpassed those of other methods. For the Leave out synthetic lethality, compared to the state-of-the-art model SLGNN, MPASL improved performance by 2.73% in AUC and 9.31% in AUPR. These results indicate that our proposed MPASL model had a stronger generalization ability and effectively enhanced the predictive performance of synthetic lethality.

**TABLE 6:** Performance comparison of MPASL and baselines.

Model	Random CV		Leave out synthetic lethality	
	AUC-ROC	AUC-PR	AUC-ROC	AUC-PR
SL^2^MF	0.7812 ± 0.0034	0.8614 ± 0.0021	0.4604 ± 0.0045	0.5002 ± 0.0061
GRSMF	0.9184 ± 0.0039	0.9361 ± 0.0024	0.6951 ± 0.0037	0.7011 ± 0.0052
DDGCN	0.8491 ± 0.0106	0.8998 ± 0.0056	0.6402 ± 0.0335	0.6352 ± 0.0334
GCATSL	0.9122 ± 0.0108	0.9175 ± 0.0078	0.7056 ± 0.0292	0.7085 ± 0.0288
KG4SL	0.9446 ± 0.0009	0.9544 ± 0.0012	0.7272 ± 0.0005	0.7623 ± 0.0003
SLGNN	0.9620 ± 0.0023	0.9703 ± 0.0019	0.8493 ± 0.0046	0.8010 ± 0.0057
MPASL	**0.9656** ± 0.0049	**0.9798** ± 0.0032	**0.8766** ± 0.0107	**0.8941** ± 0.0042

The superior predictive performance of MPASL is attributable to several key factors. First, MPASL enriches gene representations by leveraging existing SL interaction data and incorporating all relevant entities present in the KG. It effectively integrates KG hierarchy propagation and KG ripple propagation into gene embeddings enhancing the gene embeddings. MPASL also incorporates embeddings of relevant entities, weights the neighboring entities and emphasizes the most important entities, thus enriching the representation. In the process of gathering KG information, MPASL considers and blends hierarchical information and performs hierarchical contrast and aggregation, enabling the modeling of nonlinear features and higher-order interactions. This facilitates the integration of various higher-order correlation information associated with genes and neighboring entities, thereby capturing and representing the intricate interactions among gene embeddings more effectively.

### 3.3 Parameter sensitivity analysis

To gain a deeper understanding of MPASL, we researched the effect of different components on the model’s performance. First, we examined the effect of depth and width in the discrepancy contrastive layer. Then, we explored the influence of different entity embedding dimensions. Next, we studied the effect of preference set sampling size for KG ripple propagation and the effect of attention aggregator mechanism. All of the following studies were conducted based on the “Leave out synthetic lethality” setting.

#### 3.3.1 Effect of the depth and width of discrepancy contrastive layer

**FIGURE 2:**
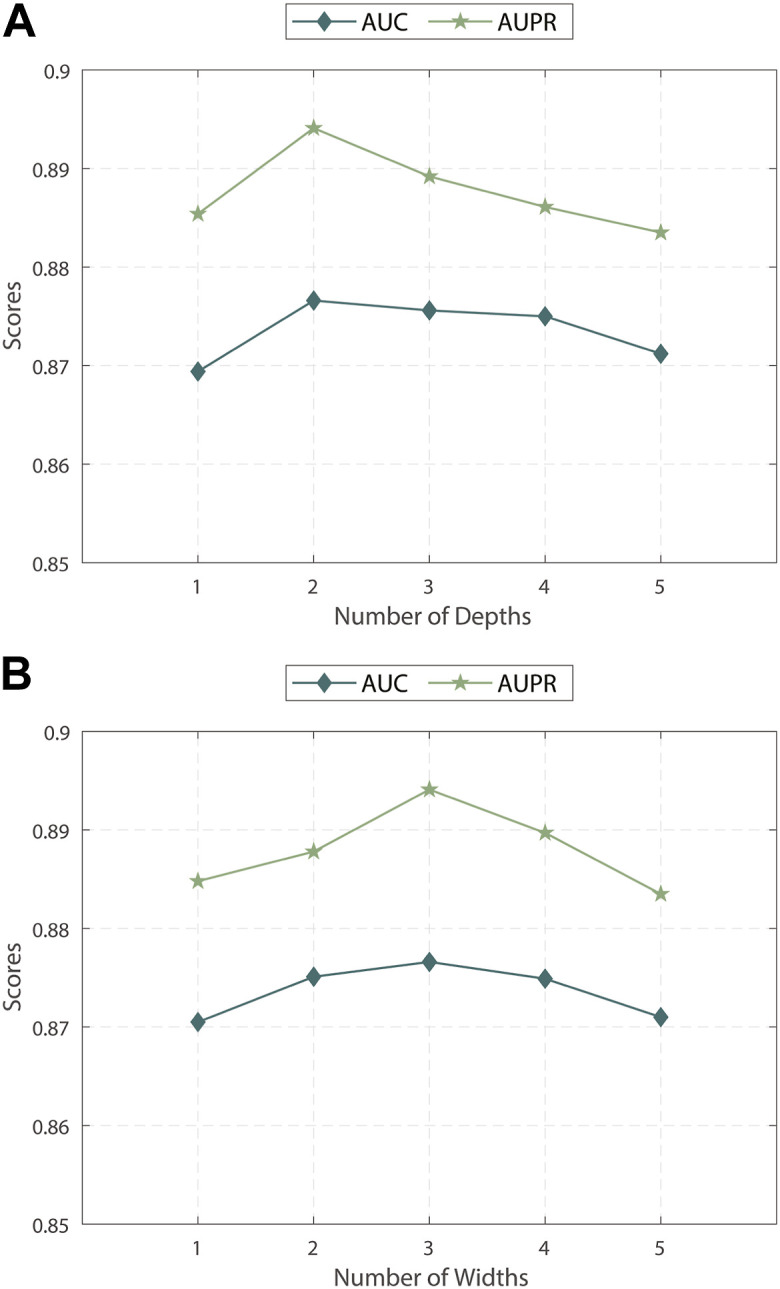
**(A)** Impact of depths of discrepancy contrastive layer **(B)** Impact of widths of discrepancy contrastive layer.

We evaluated the impact of the discrepancy contrastive layer in MPASL by varying its depth and width. As shown in Figures 2A, B, we conducted experiments within the range of {1, 2, 3, 4, 5}.

The results indicate that the performance is optimal when the depth and width are 2 and 3, respectively. Specifically, sometimes relying solely on first-order neighboring entities is insufficient to fully explore the correlations and dependencies between entities. When the depth or width is increased to 4 or 5 layers, more noise is introduced into the model. Therefore, it is necessary to balance the dependence of positive signals on distance and the noise of negative signals to find an appropriate balance in terms of depth and width allows for the exploration of potential embeddings of nodes as comprehensively as possible.

#### 3.3.2 Effect of the number of embedding dimensions

**FIGURE 3:**
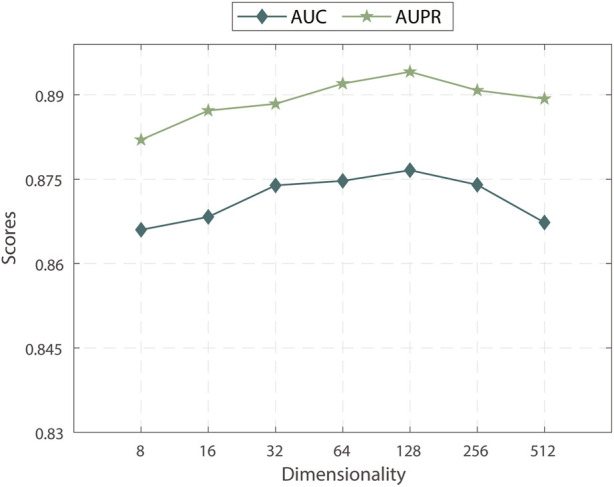
Embedding dimension of AUC and AUPR.

We explored the effects of the number of embedding dimensions on the performance of MPASL. As shown in Figure 3, we observed that the AUC and AUPR were maximized with 128 embedding dimensions. At larger numbers, the AUC and AUPR values gradually declined. In this result, it is indicates that within a particular range, increasing the embedding dimension effectively encodes more information from the KG, leading to improved performance in terms of AUC and AUPR. However, exceeding the optimal embedding dimension results in overfitting, leading to a decrease in predictive performance. Therefore, we observed an initial upward trend followed by a decline in the AUC and AUPR scores as the embedding dimension continued to increase.

Based on these findings, the key is to strike a balance when selecting the embedding dimensions for MPASL. Setting the embedding dimensions to 128 appears to be the optimal choice for capturing essential information from the KG while preserving generalization ability. This finding emphasizes the importance of appropriately adjusting the embedding dimension to achieve optimal performance.

#### 3.3.3 Effect of the KG ripple preference set size

**FIGURE 4:**
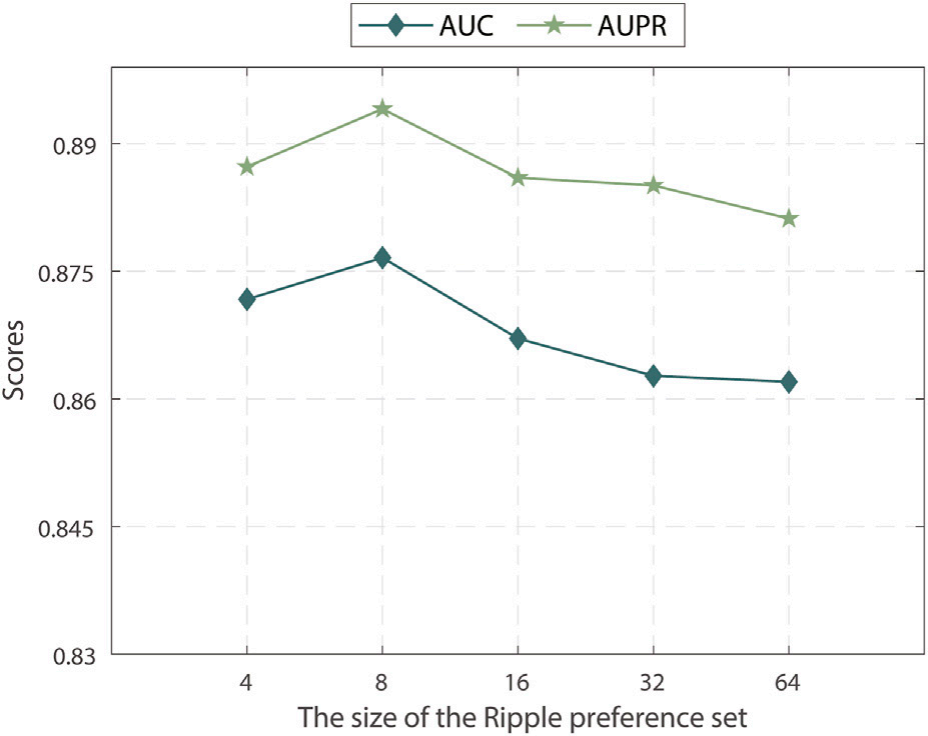
Effect of different ripple preference set size.

We investigated the impact of different sample sizes for the preference set used in the KG ripple propagation of MPASL. We varied the sample sizes within the range of {4, 8, 16, 32, 64} and analyzed their effects on the model performance. The results of the analysis are shown in Figure 4.

The results indicate that MPASL performance is optimal when the sample size of the preference set is set to 8. This means that a smaller sample size of the preference set still allows MPASL to capture sufficient information and effectively enhance gene embeddings with a limited number of known SL interactions for genes. As the preference set is further expanded, entities with lower relevance to the genes start to be included, leading to inaccurate gene embeddings and a decrease in performance. Therefore, selecting an appropriate sample size for the preference set, striking a balance between capturing sufficient relevant information as well as avoiding the inclusion of irrelevant entities, maximizes performance.

#### 3.3.4 Effect of aggregators

We evaluated the effect of different attention aggregators in MPASL: Concat, Sum, Pool, and Top-k. These are labeled as MPASL-con, MPASL-sum, MPASL-pool, and MPASL-top, respectively, in our results. As shown in Table 7, the model achieved best predictive performance when using the Top-k aggregator.

**TABLE 7:** Effect of different attention aggregators.

Aggregators	AUC-ROC	AUC-PR
MPASL-con	0.8610	0.8767
MPASL-sum	0.8627	0.8832
MPASL-pool	0.8688	0.8857
MPASL-top	**0.8766**	**0.8941**

### 3.4 Ablation study

We verified the influence of the important components on the performance of MPASL through an ablation study and designed the following four of its variants. The following study was conducted based on five-fold random cross-validation and stratified nested cross-validation settings, expressed as “Random CV” and “Leave out synthetic lethality” respectively.

(1) MPASL_w/o RP_: MPASL without the knowledge graph ripple propagation.
(2) MPASL_w/o EL(e)_: MPASL without the entity enhancement layer to update entity embedding representation.
(3) MPASL_w/o EL(att)_: MPASL without the attention aggregator to allocate weight information for entity embedding representation of different layers.
(4) MPASL_w/o R(E)_: MPASL after deleting the entity and its associated types of relationships.

We compared MPASL with several of its variants, and the results are given in Table 8. The performance achieved by the model in different cases can be summarized as follows:

• A key component of MPASL is the knowledge graph ripple propagation. We introduce the ripple propagation in order to capture the preferences of existing SL interactions to enrich the representation of genes. MPASL_w/o RP_, with the proposed KG ripple propagation removed, achieved significantly lower scores. This is because it only considered entity embedding, ignoring the set of known SL interactions of genes and the preferences of genes when aggregating entities and relationships in KG. This highlights the importance of the KG ripple propagation in our SL prediction.
• To enhance gene-specific entity information, we used the entity enhancement layer to enrich entity representations. MPASL without the entity enhancement layer, MPASL_w/o EL(e)_ was significantly outperformed by MPASL. Table 8 confirms that the entity enhancement layer improves gene-specific entity information and contributes to enhanced performance.
• Removing the attention aggregator, MPASL_w/o EL(att)_, also worsened performance compared to MPASL. compared to MPASL. Table 8 shows the importance of the attention aggregator in capturing relatively important entities and relationships from the KG, aiding in determining the weights of neighboring messages.
• The performance of MPASL_w/o R(E)_ with the removal of a particular entity and associated relationship also decreases compared to MPASL. The experimental results indicated that entities and relations in SynLethKG are helpful for SL prediction.

**TABLE 8:** Performance comparison between different variants.

Methods	Random CV		Leave out synthetic lethality	
	AUC-ROC	AUC-PR	AUC-ROC	AUC-PR
MPASL_w/o RP_	0.9395	0.9423	0.5614	0.5797
MPASL_w/o EL(e)_	0.9476	0.9556	0.8172	0.8274
MPASL_w/o EL(att)_	0.9543	0.9674	0.8271	0.8346
MPASL_w/o R(E)_	0.9616	0.9743	0.8601	0.8739
MPASL	**0.9656**	**0.9798**	**0.8766**	**0.8941**

### 3.5 Case study

To further examine the performance of MPASL, a case study was conducted using the SynlethDB dataset. The training samples included all observed known SL interactions, we used the training model to predict the SL status of unknown gene pairs. Unknown gene pairs were classified based on their prediction scores, and literature evidence was sought in the biomedical literature to support the predictions. We specifically focused on SL pairs involving the cancer gene *KRAS*. *KRAS* is one of the most widely screened genes for SL interactions and it ranks among the most frequently mutated genes in humans, particularly in cases of cancer (Downward, 2015). It is also a highly prioritized therapeutic target due to its involvement in inducing cell stasis, apoptosis, and DNA repair. In particular, we studied the top 20 SL pairs associated with *KRAS* as shown in Table 9. Among these SL gene pairs, we selected the *KRAS*-*RAD50* gene pair from the test data for further analysis. The protein encoded by the *RAD50* gene plays a crucial role in repairing DNA double-strand breaks. It interacts with *MRE11* and *NBS1* to form a complex. This complex binds to DNA and displays multiple enzymatic activities that are essential for functions such as non-homologous end joining, DNA double-strand break repair, activation of cell cycle checkpoints, maintenance of telomeres, and facilitation of meiotic recombination. This highlights the crucial role of these genes in cell growth and vitality, making it reasonable to predict their SL relationship for cancer therapeutics. In the case study, the predicted result for the *KRAS*-*RAD50* gene pair aligned with the known labels, demonstrating the accurate predictive ability of MPASL for SL pairs and emphasizing the potential therapeutic significance of the predicted *KRAS*-*RAD50* SL pair in cancer treatment.

**TABLE 9:** Top Synthetic lethality gene pairs containing KRAS predicted by MPASL.

Gene 1	Gene 2	PubMed ID	Source	Cell line
*KRAS*	*SCARF1*	19490893	GenomeRNAi	DLD-1
*KRAS*	*VDAC1*	17568748	Synlethality	Human lung cancer
*KRAS*	*IPMK*	27655641	RNAi Screen	NA
*KRAS*	*ZNF200*	24104479	Text Mining	COAD
*KRAS*	*GRK3*	24104479	Text Mining	COAD
*KRAS*	*NUDT9*	28700943	High Throughput	NA
*KRAS*	*SNRPD3*	24104479	Text Mining	COAD
*KRAS*	*RAD50*	24104479	Text Mining	COAD
*KRAS*	*PARP1*	20976469	Text Mining	cancer_D009369
*KRAS*	*PCK1*	27655641	RNAi Screen	NA
*KRAS*	*SNRPD3*	24104479	Text Mining	COAD
*KRAS*	*RPL10*	28700943	High Throughput	NA
*KRAS*	*VGLL2*	19490893	GenomeRNAi	DLD-1
*KRAS*	*TOB1*	24104479	Text Mining	COAD
*KRAS*	*PCYT2*	24104479	Text Mining	COAD
*KRAS*	*RPL7A*	24104479	Text Mining	COAD
*KRAS*	*DGKA*	27655641	RNAi Screen	NA
*KRAS*	*TRIB3*	27655641	RNAi Screen	NA
*KRAS*	*STARD10*	19490893	GenomeRNAi	DLD-1
*KRAS*	*MSL2*	19490893	GenomeRNAi	DLD-1

Table 9 consists of five columns. The first two columns represent the predicted genes that have an SL relationship, and the third column provides the PubMed ID of publications supporting the prediction. The fourth column presents the specific evidence or rationale behind each predicted SL interaction. Finally, the last column indicates the specific cell lines where the SL interaction has been observed.

**FIGURE 5:**
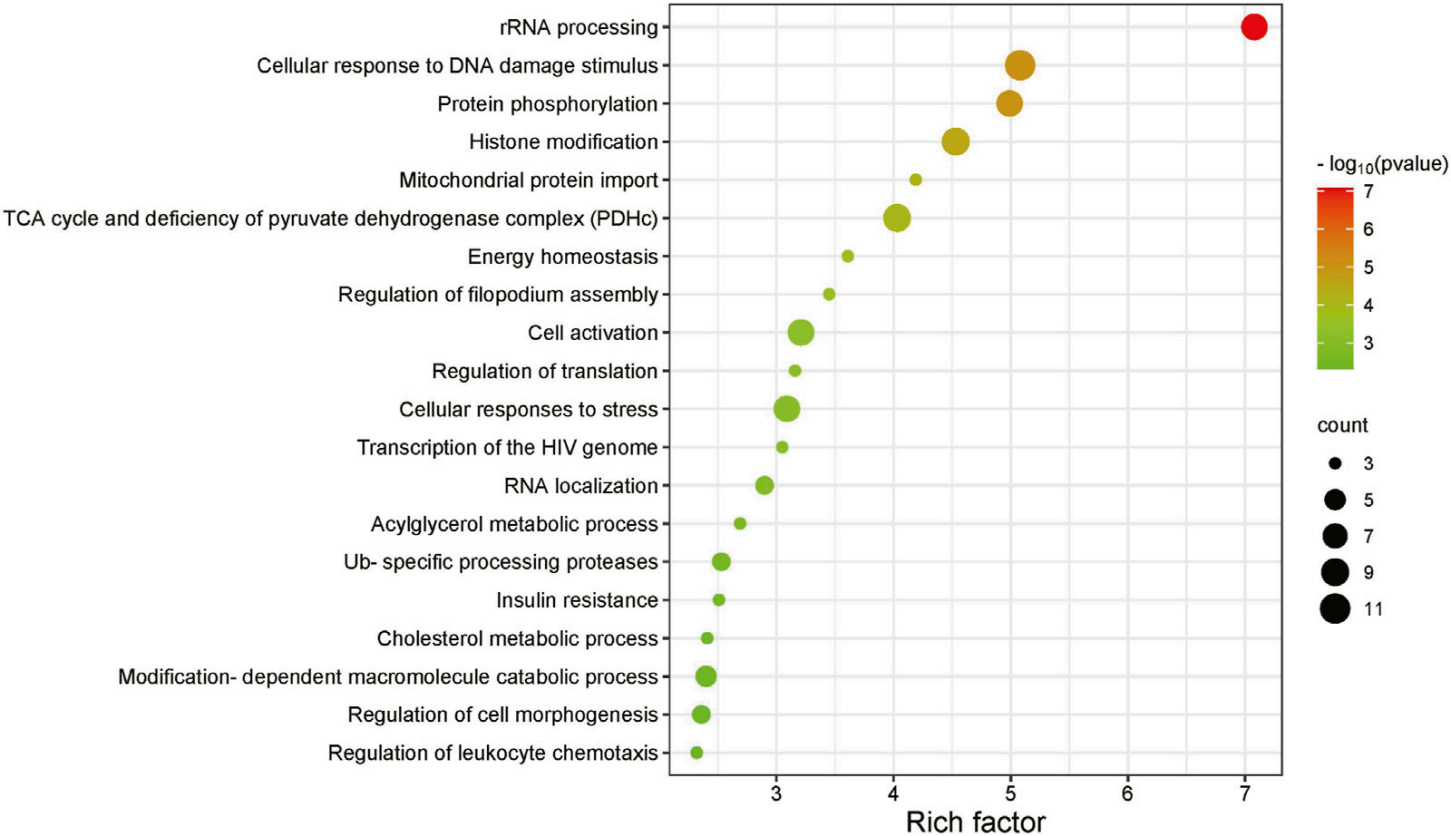
SL gene pathway enrichment analysis of KRAS.

Figure 5 illustrates the enrichment analysis results of the *KRAS* SL gene pathway. It highlights several important biological functions, including rRNA processing, protein phosphorylation, cellular response to DNA damage stimulus and histone modification. These enriched biological functions are closely associated with the expression of the *KRAS* gene and its impact on cellular proliferation or death. rRNA serves as the main component of ribosomes that synthesize proteins in cells. The proteins and enzymes encoded by genes are involved in the synthesis and processing of rRNA, regulating and promoting the maturation of rRNA. Gene expression levels and regulation can also affect the rate and efficiency of rRNA synthesis and processing. Protein phosphorylation is a vital regulatory mechanism involved in modulating diverse cellular signaling pathways. Consequently, protein kinases and phosphatases have emerged as significant targets for the development of therapeutic drugs. The cellular response to DNA damage stimulus necessitates the coordinated action of multiple DNA repair pathways. Exploiting the specific dependency of tumor cells on certain DNA repair pathways forms the basis for developing synthetic lethality-based anti-cancer research approaches. Histones contribute to maintaining DNA structure, safeguarding genetic information, and regulating gene expression, and the imbalance of histone modifications is highly correlated with tumor initiation and progression. *KRAS* plays a critical role in these processes (Manček-Keber et al., 2012; Brubaker et al., 2019). This enrichment analysis of the *KRAS* synthetic lethality gene pathway validates the predictive capability of MPASL and offers greater insight into the underlying mechanisms behind synthetic lethality.

## 4 Conclusion

In recent years, synthetic lethality has been successfully used in targeted therapy of tumors and plays an important role in targeted cancer therapy. In this study, we propose a novel SL interaction prediction model called MPASL. Based on known gene information, MPASL uses features from existing SL interaction preferences to update the gene embeddings. It also incorporates gene-entity interaction and entity-entity interaction to enrich entity embedding representation from the KG. It considers inter-layer entity comparisons and gene-related labels to better explore gene representations, stabilize the learning process on the KG, and enhance the predictive ability of the model. The experimental results show MPASL outperforms existing methods.

Pre-training strategies may help improve model performance and interpretability. Therefore, our future work will explore pre-training techniques that automatically learn features to help solve problems such as prior knowledge to extract high-quality gene embedding representations.

## Data Availability

The original contributions presented in the study are included in the article/Supplementary material, further inquiries can be directed to the corresponding author.
